# Probiotics and Prebiotics: Where Are We Going?

**DOI:** 10.3201/eid0905.030134

**Published:** 2003-05

**Authors:** Martin J. Blaser

**Affiliations:** * New York University School of Medicine, New York, New York, USA

“Probiotics and prebiotics have become part of the lexicon of food technologists,” writes Gerald W. Tannock, editor of the provocative new book, Probiotics and Prebiotics: Where Are We Going? Probiotics were defined by Fuller in 1989, as “live microbial feed supplements that beneficially affect the host animal by improving its intestinal microbial balance.” The concept of probiotics is not new, however. Approximately 100 years ago, Elie Metchnikoff, the father of immunology, investigated intestinal microbes as causative agents in aging, a process he called “autointoxication.” He believed that lactic acid–producing bacteria (such as those found in yogurt) would suppress the growth of more proteolytic, autointoxicating bacteria.

Prebiotics have been defined as “nondigestible food ingredients (usually carbohydrates) that beneficially affect the host by selectively stimulating the growth and/or activity of one or a limited number of bacteria in the colon.” Potential prebiotics have included bifidobacteria, indigenous microbes epidemiologically associated with long life and other healthful conditions.

The concept of probiotics has been developing over recent decades, and the use of prebiotics extends this idea. Although the ideas are intriguing, the central theories to be tested and the tools necessary to test them have been lacking. This book contributes substantially to addressing these difficulties. The opening chapter, by Tannock, is rich in ideas and sets the appropriate tone for the rest of the book. The other nine chapters, by 20 other authors from seven countries, address both hypotheses and specifics in state-of-the-art reviews.

The central issue in this field is the following: how can the metabolic activities of the bacterial population in the colon be manipulated to promote health? Rigorous scientific exploration of this question has been limited by two factors: the colonic biota (flora) is vast and also largely undefined. In consequence, many studies, fueled by commercial self-interest, have lacked the stringency necessary for true scientific advancement. Accordingly, a substantial portion of this book discusses the methods, current or being developed, that will help address these deficiencies. Improved methods hold the promise of better defining which bacteria are present, distinguishing how much the biota varies from person to person, and measuring how well persons respond to probiotics and prebiotics.

Another important issue is exploring the relationship of the microbial biota and the host, especially the healthy host; such microecologic studies are critical to understanding potential microbial contributions to disease. Again, development of standard methods would permit these assessments; without extensive cataloging, we cannot establish the baseline.

A third and related issue is defining conditions that might be ameliorated by probiotic or prebiotic therapies. The authors provide a long list of such diseases, including colon cancer, inflammatory bowel disease, and some less obvious candidates such as osteoporosis and atopic diseases (for example, asthma). Some researchers have hypothesized that these latter diseases result from a childhood deprived of specific pathogens, the “hygiene hypothesis.” Use of probiotics and prebiotics has been advanced as one solution to that problem.

In summary, Probiotics and Prebiotics is an important book, from which I have learned much. One deficiency, however, is the book’s remarkable absence of discussion about the role of these agents in the selection of particular bacterial populations. The focus of the book, and of the field, is largely on metabolism, but any of the anticipated therapies, especially prebiotics, will select for particular bacterial species or phenotypes. A greater focus on the biology of selection in this milieu would have been helpful. Nevertheless, this limitation does not substantially reduce the great utility of this volume to those interested in ecology, microbiology, medicine, or nutrition. This book explores a field that is out of the mainstream of human biology and medicine but deserves to be more central. For a field often marked by hyperbole because of commercially based conflicts of interest, this book is appropriately subdued and scientifically balanced. The editor and authors should be credited for their scholarly approach.

